# Multidimensional Profiles of Religiosity: Do They Matter for Gen-Xers’ Psychological and Familial Well-Being?

**DOI:** 10.1093/geroni/igaa057.1116

**Published:** 2020-12-16

**Authors:** Kent Jason Cheng, Woosang Hwang, Jeung Hyun Kim, Merril Silverstein, Maria Brown

**Affiliations:** Syracuse University, Syracuse, New York, United States

## Abstract

Although researchers have suggested that religiosity is a multidimensional construct, less is known about the long-term effects of religiosity profiles on Generation X (born between 1965 and 1980) adults’ psychological and familial wellbeing over the life courses. Thus, the goal of this study is (1) to identify unobserved profiles of young-adult Gen-Xers’ religiosity based on religious attendance, religious intensity, spirituality, and religious ideology, (2) to investigate demographic factors that predict membership in these religiosity latent classes, and (3) to examine how these profiles of religiosity predict Gen-Xers’ psychological wellbeing (self-esteem, life satisfaction, and depression) and familial wellbeing (martial satisfaction, and affectual and associational solidarity toward their aging parents) in early and middle adulthood. We selected 462 Gen-Xers from the Longitudinal Study of Generation in the 2005 (mean age = 30.25) and the 2016 waves (mean age = 41.25). In terms of data analysis, the three-step latent class analysis was conducted. We identified four religiosity profiles among young-adult Gen-Xers: strongly religious, weakly religious, literalists but not religious, religious but not literalists. Less educated single Gen-Xers were more likely to be in the strongly religious class, and less likely to be in other classes. Gen-Xers in the strongly religious class reported high scores of psychological and familial wellbeing than those in other religiosity classes. Given that the religiosity of the U.S. population has declined substantially over the past few decades, our findings indicate that religiosity is an important resource for Gen-X adults’ psychological and familial wellbeing.

